# Dynamics of p14ARF and Focal Adhesion Kinase-Mediated Autophagy in Cancer

**DOI:** 10.3390/cancers10070221

**Published:** 2018-06-29

**Authors:** Rosa Fontana, Maria Vivo

**Affiliations:** Department of Biology, University of Naples Federico II, 80126 Naples, Italy; Rosa.fontana90@gmail.com

**Keywords:** tumor suppressor, migration, cytoskeleton, tumor dormancy, anoikis, metastasis, INK4a/ARF locus, DAPK, cell spreading, EMT

## Abstract

It has been widely shown that the focal adhesion kinase (FAK) is involved in nearly every aspect of cancer, from invasion to metastasis to epithelial–mesenchymal transition and maintenance of cancer stem cells. FAK has been shown to interact with p14ARF (alternative reading frame)—a well-established tumor suppressor—and functions in the negative regulation of cancer through both p53-dependent and -independent pathways. Interestingly, both FAK and ARF (human and mouse counterpart) proteins, as well as p53, are involved in autophagy—a process of “self-digestion”—whose main function is the recycling of cellular components and quality control of proteins and organelles. In the last years, an unexpected role of p14ARF in the survival of cancer cells has been underlined in different cellular contexts, suggesting a novel pro-oncogenic function of this protein. In this review, the mechanisms whereby ARF and FAK control autophagy are presented, as well as the role of autophagy in cell migration and spreading. Integrated investigation of these cell functions is extremely important to understand the mechanism of the basis of cell transformation and migration and thus cancer development.

## 1. Introduction

The INK4A-ARF (cyclin-dependent kinase inhibitor 2A alternate reading frame) locus expresses two overlapping transcripts encoding two distinct proteins, namely, p14ARF (p19 in mouse) and p16INK4a (cyclin-dependent kinase inhibitor 2A), which share no protein sequence homology [1]. The ARF protein was initially described as a tumor suppressor that, in response to different oncogenic stimuli, by protecting p53 from proteasomal degradation, initiated a cell pathway leading to cell cycle block and/or apoptosis [2,3]. Apart from p53, ARF functionally interacts with several molecular players, such as transcription factors, stress sensors, ubiquitin ligases, protein kinases, and proteasome subunits [4,5]. This in part explains its ability to inhibit cell growth also through p53-independent activities [6]. In line with its tumor-suppressive role, ARF-deficient mice develop lymphomas, sarcomas, and adenocarcinomas, dying by 1 year of age [7]. In contrast to the compelling evidence obtained from mouse models, the importance of ARF inactivation in human cancer is less clear, with a more relevant role for p16INK4a in tumor protection [8]. ARF has been described as a nuclear/nucleolar protein. It has been shown that its accumulation in the nucleoli plays a role in stability and in the tumor suppressor functions of the protein [9]. Interestingly, ARF has been found overexpressed and/or stabilized in several cancer cells [10,11,12]. This has been correlated with its ability to perform pro-proliferative and pro-tumorigenic functions through different molecular mechanisms, at least in a subset of tumors [11,13,14,15]. Moreover, in thyroid tumors, a strong delocalization of the protein in the cytoplasm has been observed and correlated with a negative prognosis [16]. Recently, we showed that during cell spreading, p14ARF protein levels increase in the cytoplasm thanks to the activation of protein kinase C (PKC) [17]. ARF phosphorylation, by inducing ARF accumulation in the cytoplasm, stabilizes focal adhesion kinase (FAK) and allows cell spreading. The process of cell spreading/adhesion allows cells to acquire morphology and to adhere to the substrate through the coordinate polymerization and de-polymerization of actin fibers. This is accompanied by the formation of the so-called focal adhesions (FAs)—specialized subcellular structures—where integrin, activated FAK, growth factors receptors, and signaling molecules transduce outside-in and inside-out signals during the entire cell lifespan. Turnover of proteins at FAs is a highly dynamic process that regulates cell movements, cell polarity, and cell ability to invade and migrate. It is thus essential to guarantee development, survival, and differentiation. In line with this, FAK is highly expressed at both transcriptional and protein levels in human cancer, and it is involved in nearly every aspect of cancer progression [18,19,20].

Consistent with its role during cell spreading, ARF reduction in cells induces morphology defects and FAK protein level decrease followed by cell death [21]. This observation further challenged its role as tumor suppressor and demonstrates that ARF’s role within the cell is highly pleomorphic or context-dependent. While providing a halt to cell growth in some cases, it favors proliferation in others. This shows how the cell environment/status can act epistatically in directing a protein function. ARF’s involvement in autophagy [22,23,24] could, in part, account for its apparent controversial functions. Autophagy is a catabolic process, by which cells recycle proteins and even organelles during period of nutrient deprivation and upon different kinds of stress. Nutrient deprivation leads to significant increase in ARF protein levels and ARF-mediated autophagy that might protect cells from cell death [11]. It has been shown that autophagy plays a crucial role in a number of processes in different model organisms. It is involved in lifespan extension induced by dietary restriction in mammals, in delaying leaf senescence, and in developmental processes that require extensive cellular and tissue remodeling [25,26]. In recent years, autophagy has become a fundamental object of study, particularly for its role in a number of pathological conditions, such as cancer, heart disease, autoimmune diseases, and neuropathies [27,28]. While ARF involvement in autophagy has been addressed in the literature, FAK’s contribution in this process has been suggested by different approaches and appears to involve different pathways.

In this review, the mechanisms whereby ARF and FAK control autophagy are presented, as well as the role of autophagy in cell migration and spreading. Moreover, the potential involvement of ARF/FAK crosstalk in the acquisition of the metastatic traits of cancer cells are discussed in light of the new finding showing their interaction at focal adhesions.

## 2. Autophagy

In response to limited energy supplies, cells try to optimize the usage of intracellular nutrients through the induction of a literal “self-eating” (thus the name autophagy) of pre-existing components. In cancer cells, to support hyperproliferation and survival, aberrant metabolic programs are present [29], causing the production of an excess of reactive oxygen species (ROS) and waste product accumulation [30]. These inputs function as an alarm to allow eukaryotic cells to adjust metabolism to survive.

**Figure 1:**
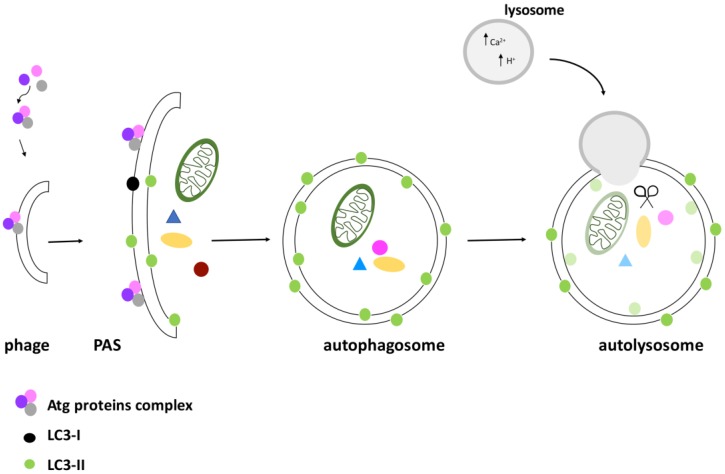
Schematic representation of autophagosome formation. The activation of the ATG (AuTophaGy-related) complex induces the formation of a pre-autophagosomal structure (PAS). The recruitment of proteins and lipids induces membrane expansion and then its closure to form the autophagosome, enveloping proteins and organelles destined for degradation. An important step in the PAS elongation is the recruitment of the protein light chain 3 (LC3), an important marker of autophagic flux. After induction of autophagy, LC3 is cleaved and turned into its activated form, LC3-I. When recruited to the autophagosome, LC3-I is converted to its lipidated form LC3-II, that can bind on both the inner and outer surface of the autophagosome. In the final stage, the autophagosome merges with the lysosome, and the cargo is completely degraded.

Three types of autophagy can be distinguished: micro-autophagy (in which proteins are degraded by lysosomes), autophagy mediated by chaperone proteins (in which specific proteins are tethered to the lysosomes), and macro-autophagy, hereafter called autophagy. In this latter case, that will be discussed in detail, a specific sequence of events leads to the formation of a vesicle with a double lipid membrane called the autophagosome, which merges with the lysosome, leading to the degradation of its contents (Figure 1).

### Molecular Control of Autophagosome Formation

**Figure 2:**
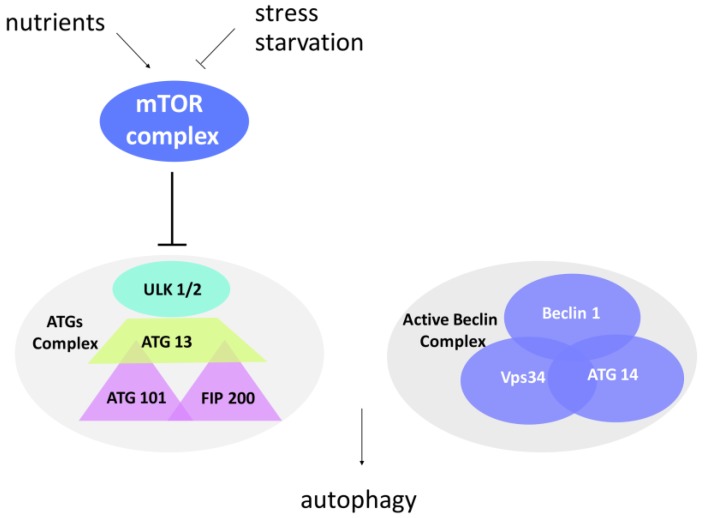
Protein complexes involved in autophagosome formation. Mammalian Target Of Rapamycin (mTOR) complex is an important sensor of stress and nutrient conditions in the cells. In response to nutrient availability, mTOR is activated, promotes cell growth, and blocks autophagy by inducing hyperphosphorylation of the ATG complex. In contrast to this, stress and starvation block the mTOR complex and induce the activation of autophagy. The ATG1 complex (uncoordinate-51-like kinase 1 (ULK1) in mammals) promotes the formation of PAS in the initiation step, while the Beclin-1 complex mediates the recruitment of ATG proteins to the phagophore in order to induce its elongation and maturation.

To avoid indiscriminate degradation, autophagy is subjected to a stringent spatiotemporal regulation. This is secured by the activity of multiprotein complexes activated only in presence of the aforementioned inputs. Nutrient-sensing pathways, growth factor signaling, and oxidative and general stress responses converge on an evolutionarily conserved protein kinase known as TOR (Target of Rapamycin) [31], placed at the crossroads between normal metabolism and autophagy. The TOR kinase, through the interaction with its binding partners, forms two complexes called mammalian TOR (mTOR) complex1 and mTOR complex2, depending on the cell and tissue types. Excess radicals or absence of nutrients inhibit mTOR complex (mTORC) kinase activity, and cell metabolism is thus switched to autophagy (Figure 2).

In presence of nutrients, the TOR complex is active and inhibits the ATG proteins (AuTophaGy-related (ATG) proteins)—the executioners of the autophagic process. Interestingly, the ATG proteins, conserved both in yeast and mammals, are functionally related to the mammalian ubiquitin system [32]. During nucleation, a so-called “dot structure” composed of a membrane portion (a phage) surrounded by ATG proteins is formed. It seems that the phage derives from the mechanical interaction of the plasmatic membrane with the endoplasmic reticulum or with the Golgi [31,33,34]. Elongation of the phage takes place through the hierarchical recruitment of ATG proteins to the membrane, thus leading to a pre-autophagosomal structure (PAS). Thanks to the ATG 13-ULK1-FIP 200 (focal adhesion kinase interaction protein of 200 kDa) complex, the PAS expands and finally closes, forming an autophagosome in which proteins or organelles are entangled (Figure 1). The ATG1 protein (ULK1 in mammals, uncoordinate-51-like kinase 1) is a serine/threonine kinase involved in the initial step of autophagy induction. FIP200 is a critical component of the ATG complex in higher eukaryotes, regulating the early steps of autophagosome formation. Differently from the other ATG proteins, FIP200 lacks an obvious sequence ortholog in yeast, although playing an essential role in autophagy. In line with this, *FIP200* gene disruption blocks autophagosome biogenesis [35,36]. This complex functions in synergy with the Beclin complex (Figure 2). The Beclin complex is composed of Beclin-1 (ATG 6), Vps34 (vacuolar protein sorting 34), and ATG 14. It mediates the recruitment of ATG proteins to the phagophore in order to induce its elongation and maturation. A fundamental role in autophagy is played by the protein MAP-LC3 (microtubule-associated protein light chain 3), that also constitutes a tool to evaluate the autophagic flux within a cell. LC3 is a cytoplasmic protein that, after induction of autophagy, is cleaved by the ATG 4 protease to generate LC3-I. The latter is then conjugated to ATG 3 and lipidized by the addition of phosphatidylethanolamine to form LC3-II. LC3-II is integrated into the membrane and is thus found on both the internal and external surfaces of the autophagosome. LC3-II is involved both in the fusion of the membranes and in the selection of the components to be degraded. Clinical studies have associated increased staining of this autophagy marker with melanoma metastases and poor survival in human breast cancer [37]. During the maturation process, the ubiquitin-binding scaffold protein p62 (also known as SQSTM1) interacts and co-localizes with LC3. It has been reported that p62 functions in selective autophagy of ubiquitinated proteins towards the autophagosome [38]. As p62 protein levels decline during autophagy induction or accumulate upon autophagy interference, p62 represents another specific marker used for monitoring the autophagic flux [39,40]. When the autophagosome completes maturation, it merges with the lysosome, forming the autolysosome, in which the action of hydrolases and proton pumps facilitate acidification of the lumen and degradation of the contents. Through lysosomal transporters and permeases, the degradation products are then brought back into the cytoplasm, where they can be recycled or used in metabolic processes [41].

## 3. Regulation of Autophagy

Regulation of autophagy occurs both at the protein level and at the transcriptional level. The key cellular regulator p53 has a role in both autophagy induction and inhibition, depending on its localization and on the type of stress [42]. Upon genotoxic stress, p53 activates a transcriptional program by transactivating genes required for autophagy induction. Among these targets there are mTOR inhibitors, such as the tuberous sclerosis complex 2 (TSC2), Sestrin 1–2, PTEN (phosphatase and tensin homolog), and the β subunits of the adenosine monophosphate-activated protein kinase (AMPK), and pro-autophagic genes, such as *Bax* and *PUMA* (p53 Up-regulated Modulator of Apoptosis), whose expression activates Beclin-1 [43,44,45]. In addition, p53 also downregulates the expression of negative regulators of autophagy, such as the Bcl-2 family members Bcl-xL and Mcl-1. Sequestering Beclin-1 is a common mechanism to block autophagy. These proteins compete with ATG 14 and Vps34 for Beclin-1 binding, thus inhibiting the formation of an active complex.

In addition to this, it has been demonstrated that, in MEF (Mouse Embryonic Fibroblast), p53 negatively regulates ARF-induced autophagy, suggesting other possible mechanisms [46]. Another pathway regulated by p53 involves the death-associated protein kinase (DAPK)—a pro-apoptotic serine/threonine kinase—which is transcriptionally induced by p53. It plays a pro-autophagic role within the cells by promoting the activation of the Beclin-1 complex through two alternative mechanisms [47,48,49]. It either phosphorylates and stabilizes Beclin-1, hindering its binding to Bcl-2, or promotes the phosphorylation of protein kinase D, which in turn activates Vps34.

In contrast to this pro-autophagy activity, several observations point to a negative regulation of autophagy by cytoplasm-localized p53 [50]. Although the mechanisms have not been completely elucidated, it appears that, in absence of stress signals, p53 can suppress the basal autophagy by either inducing ubiquitination-mediated degradation of Beclin-1 [51] or inhibition of FIP200 [52]. Another mechanism resides in p53’s ability to (indirectly) induce glycolysis and apoptosis, and thus suppress ROS production [53].

### 3.1. ARF Role in Autophagy: A Tale of Two Isoforms

The role of ARF in autophagy has been demonstrated in different cell contexts. Interestingly, signals that induce autophagy are the same as those that promote ARF upregulation. The exact role of ARF in autophagy has been challenged by the observation that a shorter isoform of the protein, designated smARF (short mitochondrial ARF), also induces autophagy in a p53-independent fashion [54]. This isoform can be generated by an additional translational initiation codon at position 48 in humans and 45 in mouse. The resulting protein lacks the N-terminal functional domain of the full-length protein, which is required for its well-known tumor suppressive functions. As with the full-length protein, the short isoform is upregulated in response to proliferative signals of cellular and viral oncogenes. Expression of smARF induces dissipation of the mitochondrial membrane in a p53- and Bcl-2-independent manner, followed by autophagy induction and caspase-independent cell death. Interestingly, the knockdown of ATG 5 or Beclin-1 reduces cell death in smARF-expressing cells, suggesting that the autophagic pathway contributes to cell death. It has been proposed that this mechanism requires smARF localization to mitochondria through the physical interaction with p32 [55]. Nevertheless, the role of smARF in autophagy is still the subject of debate. An ARF mutant, in which the internal methionine is mutated to alanine, showed that this nucleolar form, like the full-length protein and the NH_2_-terminal region alone, could still induce autophagy and caspase-independent cell death in transfected cells [56]. In this last study, it was shown that ARF can induce autophagy both in a p53-dependent and -independent manner. However, Reef and Kimchi (2008) reported that full-length ARF can induce autophagy, but only when it is strongly overexpressed in the cells, because of its localization in extra nuclear compartments [57]. In contrast, smARF can induce autophagy also when it is expressed at low levels in the cells. A recent paper published by Murphy’s group seems to clarify the role of smARF and the full-length protein in autophagy. The authors reported that p14ARF and p19ARF can induce autophagy, while smARF localizes to mitochondria where it induces mitophagy, a specific type of autophagy that selectively removes damaged or excessive mitochondria. A highly conserved domain, located within exon 2 of full-length ARF, is necessary for autophagy induction by both the human and murine proteins [46]. The involvement of smARF in mitophagy is also supported by data obtained by Grenier and colleagues (2014), who demonstrated that, both in HeLa cells and in neurons, this ARF isoform works as an upstream regulator of PINK1 and Parkin, two important proteins that mediate the priming of damaged mitochondria for mitophagy [58]. Interestingly, these authors reported that full-length ARF is not able to induce mitophagy, at least in their experimental conditions. In contrast with this finding, there is the observation that smARF expression can also inhibit mitophagy [59]. Indeed, it has been shown that in response to different stresses, such as hypoxia and nutrient deprivation, the kinase Jnk2 (c-Jun NH_2_-terminal kinase) promotes the ubiquitination-dependent proteasomal degradation of endogenous smARF. In particular, they observed that the loss of Jnk2 causes a strong increase of smARF endogenous protein levels. This led to an enhancement of autophagy and thus degradation of the adaptor p62, a mitophagy inducer. In conclusion, it appears that endogenous smARF can regulate the balance between autophagy and mitophagy by two distinct mechanisms: promotion of steady-state autophagy and suppression of stress-induced mitophagy.

This scenario has been further enriched by the observation that ARF physically interacts with the Bcl2 family member Bcl-xL at the mitochondria [23]. Bcl-xL interacts with Beclin-1 and negatively regulates the kinase activity of the Vps34/Beclin-1 complex [45,60]. It is important to highlight that it is difficult to detect the Beclin-1/Bcl-xL complex by co-immunoprecipitation, and the fraction of ARF that interacts with Bcl-xL in cells is very low. By virtue of physical interaction with Bcl-xL, ARF hampers its binding with Beclin-1, thus exerting pro-autophagy functions.

These data underline how the molecular mechanism through which ARF can promote autophagy in the cells follows routes that are not yet entirely addressed, and, in some cases, apparently in contrast with each other. The controversial results obtained by different groups in the field could be partly due to differences in the cell and experimental contexts. Comparison between the full-length and short ARF isoforms is further complicated by the different stabilities of the two proteins. Moreover, as autophagy is a stress sensor mechanism, its levels constantly fluctuate as cell conditions change. Another source of complexity comes from the observation that the molecular pathways differ between basal and starvation-induced autophagy. The observation that ARF, similar to ATG proteins, localizes to FAs during cell spreading opens the way to the possibility that its function relies on the activation of autophagy at specific sites within the cell. Moreover, the regulation of ARF by protein kinase C (PKC) suggests that, since its tumor suppressor ability can switch off [17,61], its function in autophagy can be regulated by post-translational modifications as well.

### 3.2. FAK Control of Autophagy: Regulation in Time and Space

The regulatory circuits governing autophagy analyzed so far are entangled with inside-out and outside-in signals transduced by the ECM and cytoskeleton. Loss of ECM–integrin engagement results in induced autophagy in several cellular models [62,63]. Furthermore, the basal level of autophagy regulates cell spreading by promoting cell protrusion extension [64] and is required for focal adhesion turnover and cell migration [65]. Interestingly, upon loss of ECM contact, the absence of nutrients does not per se induce autophagy, but rather a signaling mechanism that, “sensing” the cell environment, transduces the need for an alternative energy supply mechanism to the cell [66]. Cell interaction with the extracellular matrix results in integrin-mediated tyrosine phosphorylation and recruitment of FAK to focal adhesions [67]. Intramolecular interaction between the FERM (Four-point-one, Ezrin, Radixin, Moesin) and the catalytic domains of FAK results in a conformationally closed structure in which both auto-phosphorylation and Src recruitment are blocked [68]. Upon integrin and ECM interaction, a conformational change of the FERM domain occurs. This leads to the auto-phosphorylation of Tyr 397 followed by Src binding. Src in turn mediates phosphorylation of FAK on Tyr 576 and 577, ultimately leading to full activation of FAK’s catalytic ability. Once activated, FAK phosphorylates numerous proteins, providing a scaffold on which focal adhesion is organized. It has been observed how plating cells on different substrates modulates their autophagic flux, both in the basal condition and upon starvation [69]. In agreement with its involvement in the process, FAK downregulation by RNA interference decreases autophagy. This is interesting also in light of the physiological role of this pathway in cell homeostasis maintenance and development.

**Figure 3:**
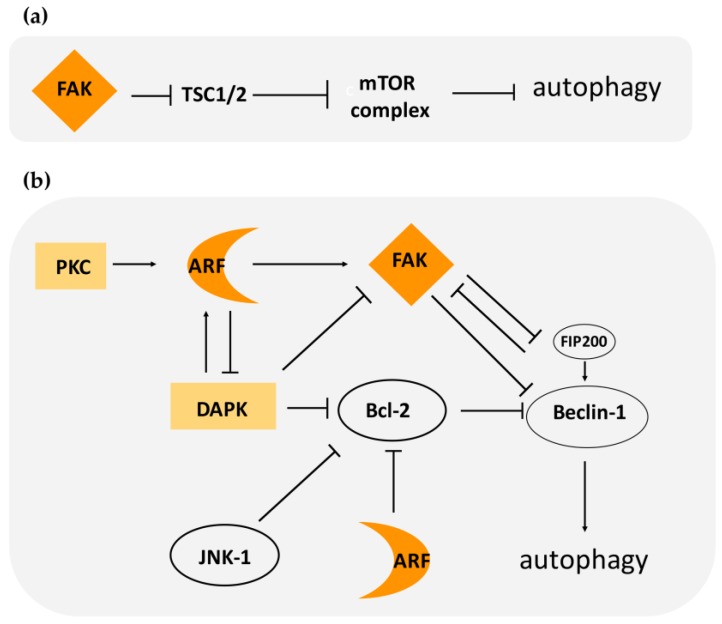
Pathways of focal adhesion kinase (FAK) and alternate reading frame (ARF) mediated regulation of autophagy. (**a**) FAK can induce activation of the mTOR complex by blocking tuberous sclerosis complex (TSC)1/2. (**b**) FAK can block the activation of the Beclin-1 complex by sequestering FIP200 or through direct phosphorylation of Beclin-1. Bcl-2/Bcl- XL sequester Beclin-1, inducing autophagy inhibition. ARF can either activate FAK or block Bcl family members. In light-orange boxes are represented proteins (protein kinase C (PKC) and death-associated protein kinase (DAPK)) that positively regulate ARF stability. DAPK can promote autophagy by blocking Bcl2 and inducing the Beclin-1 complex.

At the molecular level, FAK involvement in autophagy relies on its ability to activate the mTOR complex. In response to cell–matrix adhesion, FAK is activated and maintains mTORC in an active state through suppression of TSC2, an upstream mTOR negative regulator (Figure 3). It has also been reported that FAK can induce the activation of mTOR through AKT [70,71].

Another molecular target of FAK is Beclin-1. FAK’s ability to phosphorylate Beclin-1 on Tyr 233 leads to the inhibition of a functional Beclin complex [72]. Thus, FAK can inhibit autophagy through different routes: by activating mTORC and thus normal cellular metabolism, and through the inhibition of autophagosome formation (Beclin complex) (Figure 3).

Early evidence for a functional link between autophagy and focal adhesions derives from the identification of the molecular interaction between FIP200 and FAK. FIP200 has been shown to co-localize with FAK at focal adhesions and to inhibit FAK kinase activity and its autophosphorylation. The formation of a FIP/FAK complex is strongly favored in suspended cells, indicating that their association correlates with FAK inactivation upon cell detachment [73]. FAK sequestering of FIP200 has been proposed as a mechanism through which FAK interferes with ATG complex formation [74]. Intriguingly, it has also been reported that FIP200, by inhibiting the TSC1–TSC2 complex, can release their negative function on mTOR, thus blocking autophagy [75]. This observation supports the idea that the cellular environment has a clear impact on the fate of the molecular players who act in autophagy. Indeed, in the presence of certain stimuli, a protein may be an inhibitor of autophagy, whereas in a different condition the same protein has the opposite function.

Another possible mechanism through which FAK can regulate autophagy is through the inhibition of p53. Interestingly, it has been shown that FAK promotes cell survival by facilitating p53 ubiquitination and proteasome-mediated degradation [76,77]. In support of this, FAK accumulates in the nucleus within 30 min after loss of adhesion, and this is accompanied by loss of FAK from cellular focal contacts [76]. On this basis, we could speculate that FAK localization in the nucleus, by inhibiting autophagy, could favor the formation of FA complexes and thus trigger cell spreading. It would be interesting to analyze how FAK’s role in autophagy depends on the nature of the induced signal, such as the need to adhere to the substrate in a given condition, or its response to hypoxia or a free radical increase inside the cell. Similarly, it would be interesting to compare the relations between FAK and the pathways involved in starvation/stress-induced autophagy and basal autophagy.

In addition to this hypothetical nuclear FAK function, it has been reported that FAK at FAs has a role in determining the fate of FA proteins. Recruitment of some ATG proteins at FA, and their co-localization with FAK and Src, has been observed and correlated with autophagy induction and cell survival. In particular, in squamous cell carcinoma (SCC), the absence of FAK or alteration in the Src/FAK pathway induces an acceleration of the autophagy rate that causes degradation of FAK’s binding partners. In particular, in these conditions, Src and Ret translocation from FAs to the cytoplasm and their autophagosome-mediated degradation has been observed. The excess of untethered Src proteins to FAs causes apoptosis. Thus, its autophagy-mediated degradation occurs as a means to ensure cell survival [78,79]. Src translocation from FAs to the autophagosome occurs through its binding with c-Cbl, an E3 ubiquitin ligase, which interacts with LC3. The accelerated rate of autophagy observed in these conditions is necessary to block the programmed cell death caused by the loss of anchorage-dependent attachment to the extracellular matrix, or anoikis, as reported for SCC, a treatment refractory malignancy [80]. This supports the idea that autophagy can protect matrix-detached epithelial cells. These studies highlight FAK’s role in protecting Src from autophagy-mediated degradation. Further insights into this mechanism comes from the observations that the autophagy protein Ambra 1 controls spatial localization of active Src in cancer cells [81]. In particular, in SCC FAK^−/−^, Ambra 1 regulates translocation of active Src from focal adhesions to the autophagosome through its binding partners dynactin and IFITM3 (Interferon-Induced Transmembrane Protein 3). Src trafficking to autophagic structures is also achieved through Eps8-induced actin rearrangements [82]. Eps8 is an actin regulatory scaffold protein whose expression is increased in SCC and is associated with the aggressive cancer phenotype. In FAK-proficient SCC cells, active Src becomes tethered at FAs, where both Eps8 and Ambra 1 are recruited and required for FAK-dependent cancer processes like adhesion, invasion, polarization, and 3D proliferation [81].

It has been shown that FAK, through Src signaling, can induce activation of Rho signaling, that in turns mediates ERK2/MAP kinase activation [83]. Among ERK2/MAP kinase targets, there is the c-Jun NH_2_-terminal kinase (JNK). The phosphorylated and thus active form of JNK has been correlated with increased cell invasion and migration. Interestingly, JNK kinase can phosphorylate Bcl2, thus abolishing Bcl-2-mediated repression of Beclin-1 and promoting autophagy upon starvation (Figure 3). This suggests that, in certain circumstances, FAK expression can have a positive effect on autophagy induction, at least in some cellular contexts [84].

Taken together, these results led to the hypothesis that FAK’s role in autophagy can be important not only in the global activation or inhibition of autophagy, but in the fluctuation between different cellular statuses, characterized by either high or low levels of autophagy. FA turnover needs to be timely regulated in a dynamic fashion by means of protein levels/activity or through transcriptional control of autophagy genes. A slow turnover of FAs can endow the cell the time to adhere to the substrate and to acquire a given morphology or polarity. In contrast, increased autophagy-mediated degradation could promote migration and invasion by allowing more cell movements. Interestingly, FAK could selectively protect its binding partners from autophagic degradation at specific subcellular locations within the cell, as seen with Src, thus regulating FA turnover.

## 4. ARF–FAK Cross-Talk

**Figure 4:**
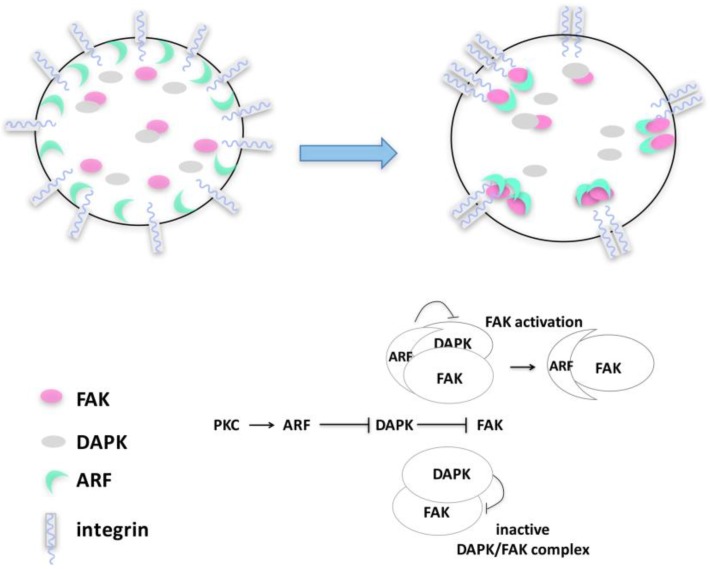
p14ARF–FAK cross-talk upon cytoskeleton remodeling. Upon PKC activation during the initial steps of cell spreading, p14ARF protein accumulates in the cytoplasm and is recruited at focal adhesions where it interacts with FAK, inducing its accumulation. Upon substrate adhesion, a p14ARF/FAK/DAPK protein complex is detected in the cytoplasm, in which ARF induces FAK stabilization, protecting it from DAPK degradation.

By monitoring spreading in different tumor cells, we observed that p14ARF is recruited to sites of active actin polymerization, in particular to filopodia [21]. Here, a functional interaction with active FAK takes place in a precise time window. Consistently, p14ARF depletion causes a failure in the acquisition and maintenance of cell morphology. We postulate that, through physical interaction, ARF can exert a protective role on FAK upon cell detachment and adhesion (Figure 4). By aiding cytoskeleton assembly during spreading, p14ARF protects cells from anoikis, blocking DAPK-dependent apoptosis.

It has been shown that FAK activation can be counteracted by DAP kinase expression by disrupting signal transduction between integrin and FAK upon ECM interaction [85,86,87]. In line with this, p14ARF expression prevents DAPK-mediated FAK degradation [21]. During cell spreading, a complex composed of DAPK, FAK, and p14ARF can be detectable (Figure 4). Our data led us to conceive a model in which, during cytoskeleton remodeling, p14ARF protein levels increase in the cytoplasm thanks to PKC-mediated stabilization. In this time window, DAPK would interact with FAK, preventing its improper activation. The increase of ARF protein levels gradually interferes with this mechanism, hampering DAPK-mediated inhibition of FAK (Figure 4), maybe by competing with DAPK in FAK binding. This is associated with an increased proliferation rate mirroring increased FAK phosphorylation. Insights within the molecular mechanism at the basis of ARF/FAK functional interaction come from the observation that the ARF protein domain required for both pFAK stabilization and cell survival is located in a specific region in the C-terminal of the protein. Interestingly, this domain plays an established role in autophagy [46], as previously described, suggesting that ARF’s ability to regulate autophagy could be involved in this mechanism.

In addition to this data, another intersection between ARF functions and FAK activation is the observation that ARF can mediate FAK sumoylation [21], as reported for other ARF binding partners [88]. It has been reported that sumoylated FAK has a strong autocatalytic activity and localizes to the nucleus [89]. An attractive hypothesis is that, by mediating FAK sumoylation, ARF induces or sequesters FAK to the nucleus, thus preventing its degradation. Cytoplasmic-nuclear translocation has been demonstrated for other focal adhesion proteins [67], and also for ARF. The nuclear ARF–FAK complex could thus function as “storage” of highly active proteins that, upon certain environmental conditions, aids the cell to spread. This could be an interesting hypothesis in the process of cancer dissemination through the body, allowing the successful implantation of cells in the metastatic niche. This could be achieved, at least in part, through the inhibition of p53-mediated apoptosis by interfering with the ARF-p53-MDM2 (Mouse Double Minute 2) circuit [90]. Moreover, by targeting MDM2 to p53 in the nucleus, FAK could prevent ARF degradation by MDM2 [91], increasing ARF-mediated autophagy induction and survival. Further supporting this hypothesis, it has been shown that nuclear-localized FAK binds numerous transcription factors in both physiological and pathological contexts [68]. It is interesting to underline that, upon genetic stress, FAK also localizes to nucleoli, and this is associated with its ability to confer anchorage-independent growth of cancer cells [92]. As ARF localization to nucleoli has been correlated with its ability to block cell proliferation and ribosomal RNA processing [93], it would be interesting to analyze ARF’s role in nucleolar FAK functions and recruitment. Moreover, given the role of ARF phosphorylation in its cellular localization, it would be worth evaluating its role in this process [17,61]. ARF activity in the cytoplasm appears to be timely regulated by a PKC-mediated mechanism. Upon cell spreading induction, thus during the de novo formation of FAs, PKC is activated and ARF levels increase. An ARF mutant that mimics the phosphorylated status of the protein rescues both spreading and FAK tyrosine phosphorylation defects of ARF-depleted cells. It is thus possible to envisage that in absence of ARF (or in absence of PKC phosphorylation), FAK could be sequestered by FIP200 and kept in an inactive state (Figure 3). This is supported by the observation that that increased expression of FIP200 inhibits both FAK autophosphorylation and cellular migration [94,95]. These interactions could thus function to coordinate cell proliferation rate, autophagy, and migration. FAK activation is followed by ERK phosphorylation and the transduction of a pro-proliferative signals to the cell nucleus. Also, AMPK activation induces autophagy but negatively affects cell migration in an Ulk1- and FIP200-dependent manner in several tumor cells [96]. Interestingly, the ARF phosphomimetic mutant is impaired in cancer cell growth suppression [61], but it stimulates cell survival in HeLa cells [17].

Interestingly, inhibition of autophagy by ATG 5 or ATG 7 depletion decreases cells’ ability to spread and to form protrusions, similar to ARF-depleted cells [21]. In agreement with these observations, we found a reduction of autophagic flux upon ARF silencing. It would be interesting to analyze the role of ARF-mediated autophagy on cytoskeleton dynamics. As cell death impairs further analysis, it would be interesting to analyze the effect of ARF depletion on cell migration in anoikis-resistant cell lines. We also observed that ARF depletion has a negative effect on global tyrosine phosphorylation of FAK, thus suggesting that high levels of FAK could be associated with increased malignancy of ARF-expressing tumor cells. It would be interesting to understand if, and which, tyrosine phosphorylation is increased by ARF, or if it is the ratio between FAK and phosphorylated FAK that has a role in cell migration and (or) in cell proliferation. It has to be underlined that, as ARF-induced FAK stabilization also results in an increase in cell viability, we could not discriminate if ARF-mediated autophagy affects cell motility independently of cell proliferation.

## 5. Conclusions: Autophagy and Cancer Evolution

Many studies suggest a dual role of autophagy, involved both in tumor suppression and in survival. It has been reported that genetic loss of *ATG* genes makes cells “tumor prone”. Nevertheless, many studies and clinical trials indicate that established tumor cells use autophagy as a crucial survival pathway [22,97,98,99]. This duality can be explained if we consider autophagy as a safety metabolic plan for the cells. A normal growing cell, thanks to a functional autophagy pathway, gets rid of free radicals and damaged organelles, thus avoiding genotoxic stress and preserving integrity of the genome. At the same time, if induction of autophagy occurs in a cell that has already received insults that provoked aberrant transformation, the instauration of such a pathway can fuel cancer development by avoiding apoptosis and/or anoikis and allowing tumor dissemination within the body.

Recent reports showed how autophagy is required for the motility and invasion of highly metastatic tumor cells. Although they do not depend on autophagy for cell proliferation and survival, inhibition of autophagy in a breast cancer model reduces lung and liver metastases in vivo. This is due to the inability of cancer cells to escape from the primary tumor site [100]. The authors showed that oncogenic Src mediates the interaction between paxillin and LC3. This is followed by autophagic degradation of paxillin that promotes rapid FA turnover and cellular migration. These results highlight the role of autophagy in the process of cancer evolution, the multistep process that, from an initial mutated cell, leads to a metastatic cell that can efficiently colonize secondary sites within the body. Metastasis is one of the clinical parameters with a strong negative influence on the prognosis of cancer patients. Its detection in patients is usually associated with resistance to chemotherapeutic treatments and higher post-treatment recurrence states. The ability to form metastasis is accompanied by several changes not only in cellular metabolism but also in the cell architecture. One cancer cell feature is the alteration of the adhesion process, as transformed cells need to lose contact inhibition to continue growth. In addition, alterations in adhesion properties can have the effect of favoring cell migration as a result of unbalanced actin dynamics. Despite its malignancy, the metastatic process is highly inefficient; it is estimated that less than 0.5% of cells entering the circulatory system form metastasis [101]. Most cells die in non-permissive tissues, while others enter a dormant state in the peripheral niche, either at the single-cell or micro-metastasis stage. When appropriate growth conditions arise, these cells exit the dormant state to form metastasis. Despite this low efficiency, this process causes about the 90% of cancer deaths. Some studies suggested that autophagy may protect tumor cells from anoikis by facilitating tumor dormancy [102]. In this case, autophagy can mediate survival during metabolic stress, arising upon extravasation and migration to the metastatic niche. This can be accomplished because autophagy confers to the cell “extra-time” for recovery, allowing re-adaptation to a new environment. A similar cancer-prone effect has been described in cellular protection from genotoxic stress caused by chemotherapy agents. By promoting the development of chemotherapy-resistant cancer cells, autophagy allows tumor relapse. In addition to these mechanisms, by deciphering the fate of proteins at FA, increased autophagy can be considered as a tool to shape the cellular cytoskeleton and endorse cells with pro-migration morphology.

It has been widely reported that PKC-mediated phosphorylation regulates composition and turnover of focal adhesions. In particular, cellular motility and invasion, two of the worst signals of cancer progression, are sustained by assembly and disassembly of actin fibers, a highly dynamic phenomenon [103,104,105]. The involvement of FAK in tumor progression has thus gained recognition as potential therapeutic targets for the treatment of various malignancies. A deeper understanding of the FAK and ARF functions, both in physiological and pathological contexts, may provide useful information about the environmental cues that determine ARF functions as a tumor suppressor or tumor promoter. These events could cooperate to mediate survival and/or tumor dormancy in otherwise non-permissive conditions, further underscoring the need for a careful re-evaluation of the status and role of p14ARF in human cancer.
